# A Case of Cytomegalovirus-Induced Oral Ulcer in an Older Adult Patient with Nephrotic Syndrome due to Membranous Nephropathy

**DOI:** 10.1155/2020/8843816

**Published:** 2020-09-29

**Authors:** Shinichi Sato, Masato Takahashi, Tetsu Takahashi

**Affiliations:** ^1^Oral and Maxillofacial Surgery, Japan Community Health Care Organization Sendai Hospital, 3-16-1 Tsutsumimachi, Aoba-ku, Sendai 981-8501, Japan; ^2^Division of Oral and Maxillofacial Surgery, Tohoku University Graduate School of Dentistry, 4-1, Seiryo-machi, Aoba-Ku, Sendai 980-8575, Japan

## Abstract

We report a case of cytomegalovirus- (CMV-) induced buccal ulcer in a patient with nephrotic syndrome. An 82-year-old man with membranous nephropathy was on immunosuppressive therapy presented with an ulcer in the oral cavity and was hospitalized. Intraoral examination revealed an inflamed and painful ulcer on the left buccal mucosa. Blood test results showed CMV positivity, and histopathological examination confirmed the diagnosis. Anti-CMV therapy (ganciclovir) was initiated from the third day of hospitalization. However, he developed dyspnea on the 14th day. Computed tomography images of the chest revealed the presence of ground-glass opacities, and noninvasive positive pressure ventilation was initiated under the provisional diagnosis of pneumocystis pneumonia caused by ganciclovir-associated myelosuppression and/or steroid-induced immunocompromised state. The patient died of pneumocystis pneumonia on the 21st day. The patient had received immunosuppressive therapy for renal dysfunction. Immunocompromised patients with CMV infection should be treated with caution, as drugs for CMV may themselves cause myelosuppression, deteriorating the prognosis of the patient.

## 1. Introduction

Cytomegalovirus (CMV) is a genus of viruses of the order Herpesvirales, which is estimated to infect approximately 90% of adults at some point in their lives. CMV infection is usually subclinical and can persist in this manner for the entire life span of an infected individual [1–3]. It is also an opportunistic pathogen [2] and can affect multiple organs, leading to pneumonia, peptic ulcers, retinitis, and/or encephalitis in immunocompromised hosts, including patients who have undergone allogeneic hematopoietic stem cell transplantation [4, 5], allogeneic bone marrow transplantation [6, 7], and/or organ transplantation [8] and patients infected with the human immunodeficiency virus (HIV) [9].

To date, however, few reports have documented the manifestations of CMV infection in the oral cavity [10–12]. Here, we report a case of CMV infection of the buccal mucosa in an immunocompromised patient.

## 2. Case Presentation

An 82-year-old man was referred to our hospital from another clinic on June 11, 2019; at that time, he had low total protein (4.8 g/dL)/albumin levels (1.6 g/dL) and high protein-creatinine ratio (7.3) and was thus admitted to our department of nephrology. His medical history revealed frequent tonsillitis in childhood and two episodes of hemorrhagic gastric ulcers at the age of 76 years. He was not positive for HIV, and no family history of renal failure or history of consanguineous marriage was reported.

He was diagnosed with membranous nephropathy based on the findings of a renal biopsy and was treated according to the standard treatment protocol [13], which included prednisolone (30 mg) and cyclosporine A (100 mg). Steroid pulse therapy was not initiated, and he was discharged from the hospital on July 9 (Table 1).

On July 26, he started experiencing pain and difficulty in chewing due to pain in the entire oral cavity, and on August 5, he was admitted to our hospital on an emergency basis due to the presence of a severely painful lesion in the mouth.

At the time of admission on August 5, 2019, his height and weight were 160 cm and 62.9 kg, respectively. Results of blood tests revealed the following: albumin concentration, 1.3 g/dL; C-reactive protein concentration, 1.39 mg/dL; total protein concentration, 4.1 g/dL; platelet count, 14.7 × 10^4^/*μ*L; and leukocyte count, 35.9 × 10^2^/mm^3^. The results of further antibody tests revealed that he was positive for CMVpp65 antigens (C10, C11) 546/434. His *β*-D glucan concentration was 40.2 pg/mL, suggesting a strong possibility of CMV infection.

**Figure 1:**
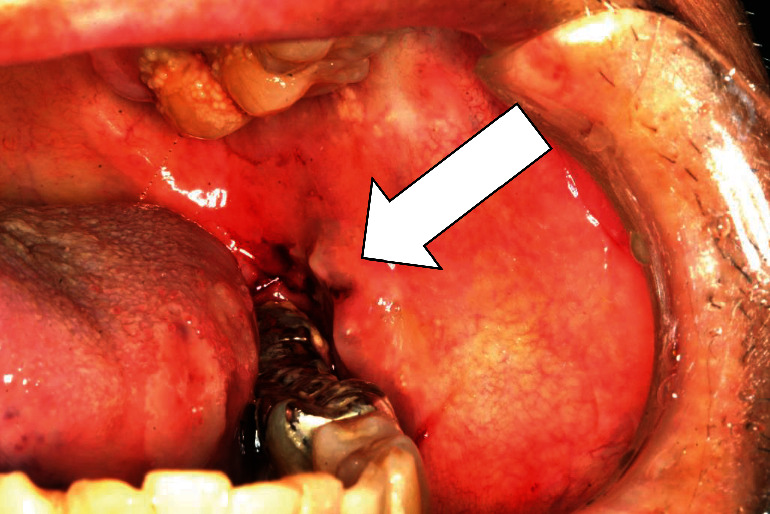
Oral findings in the patient at the initial visit. Ulcers are visible on the left buccal mucosa. Moreover, an erosion is seen on the tongue. Open arrow: cytomegalovirus-associated ulcer.

On August 6, 2019, the patient presented with a painful intraoral ulcer; intraoral examination revealed ulcers on the left buccal mucosa and generalized inflammation of the gingiva. Moreover, erosion was observed on the left side of the tongue (Figure 1). No ulceration or erosion was observed on the right buccal mucosa or the right side of the tongue.

A clinical diagnosis of ulcer on the left buccal mucosa was made, and the attending physician requested a biopsy of the ulcer for a definitive diagnosis. An incisional biopsy was performed, and the specimen was sent for a pathological examination.

**Figure 2:**
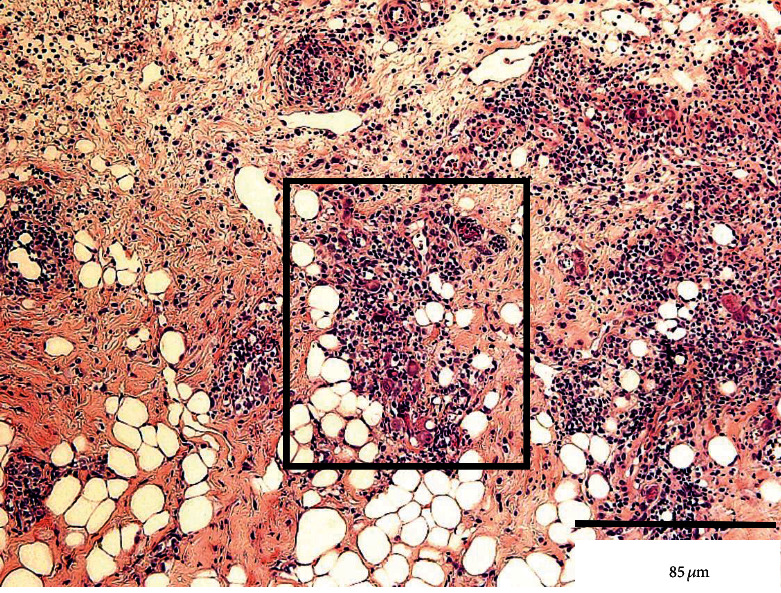
Histopathological findings of the specimen obtained from the biopsy of the ulcer. The black box shows vascular endothelial cells containing enlarged nuclei with intranuclear inclusions (hematoxylin-eosin staining). Bar: 85 *μ*m.

**Figure 3:**
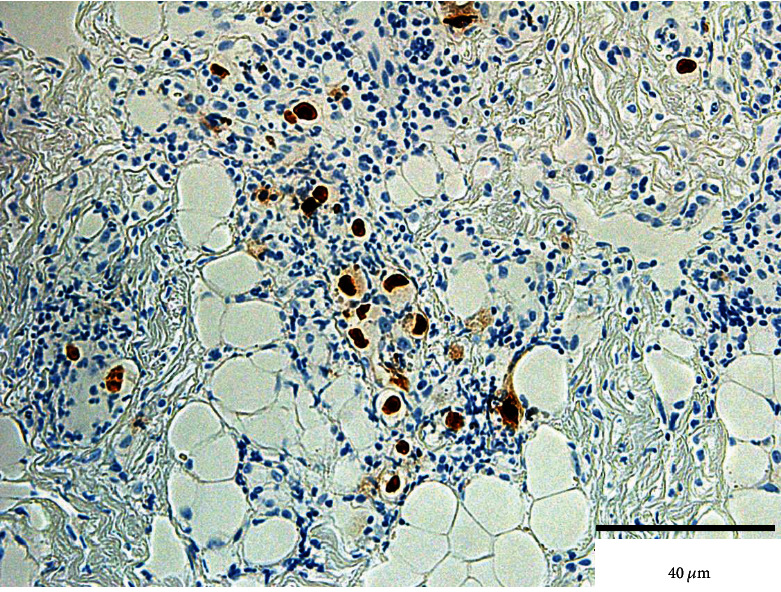
Findings from immunohistochemical staining of the specimen obtained from ulcer biopsy. Anticytomegalovirus antibodies are detected in the nuclei of infected cells. Bar: 40 *μ*m.

Histopathological findings revealed a prominent ulcerative lesion containing numerous CMV-positive vascular endothelial cells scattered at the fundus of the ulcer (Figures 2 and 3). Based on these findings, the diagnosis of CMV infection was confirmed.

The pain persisted even after admission, and the patient was prescribed an azulene sodium sulfonate gargle containing 4% xylocaine. A 10-day course of anti-CMV medication (ganciclovir) was initiated on August 7, 2019.

**Figure 4:**
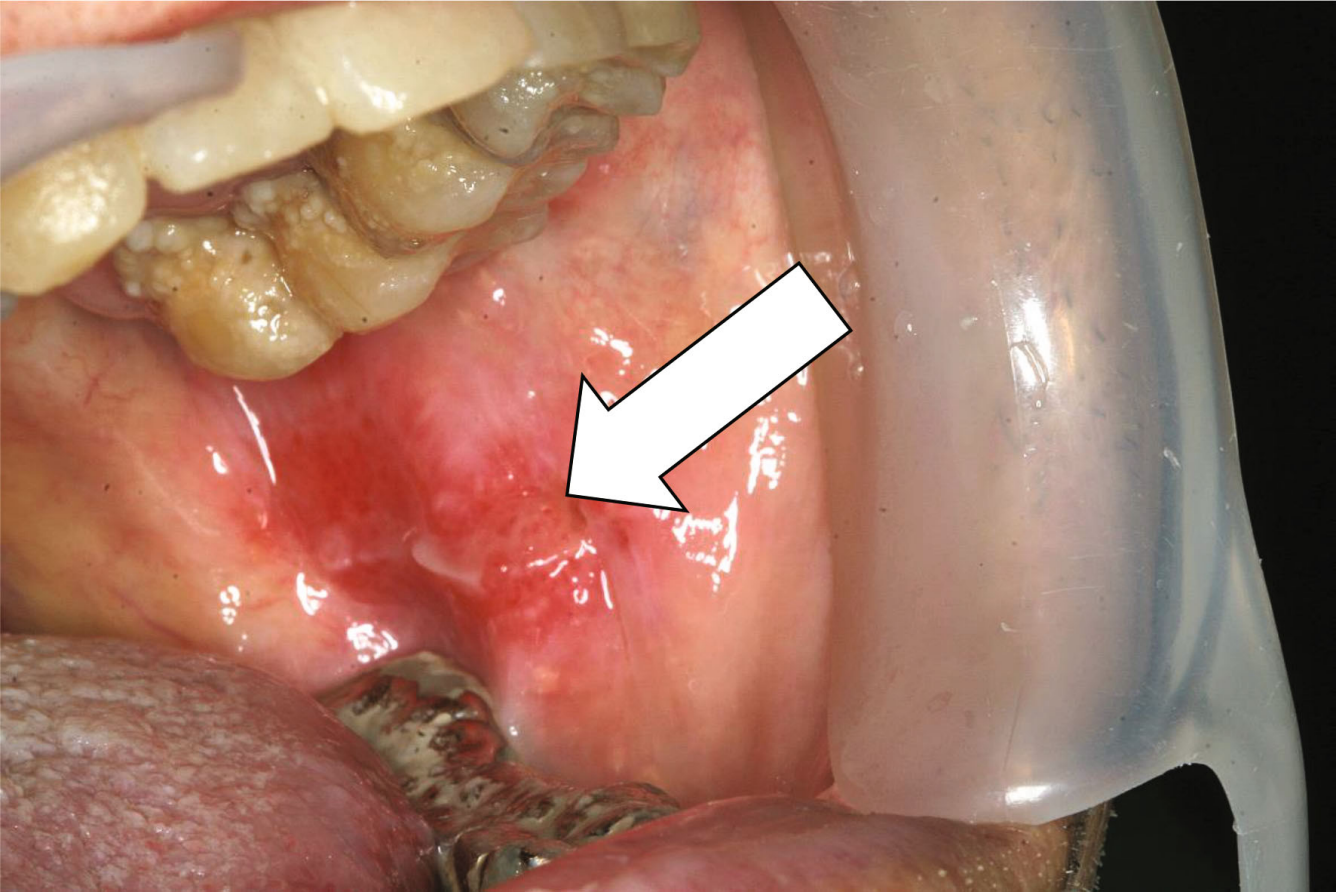
Oral findings on day 9 after initiation of ganciclovir therapy. A persistent ulcer on the left buccal mucosa is visible, with reduced signs of inflammation. Open arrow: cytomegalovirus-associated ulcer.

**Figure 5:**
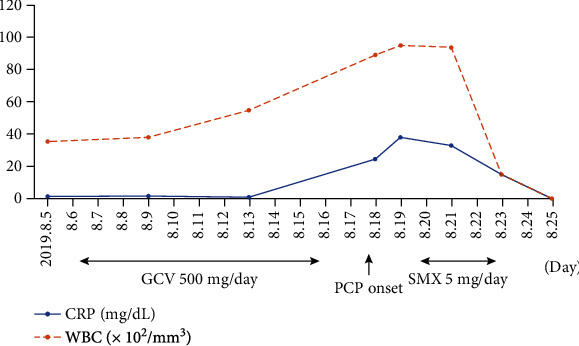
CRP and WBC levels and the administered medications during hospitalization are shown. CRP: C-reactive protein; WBC: leukocytes; GCV: ganciclovir; PCP: pneumocystis pneumonia; SMX: sulfamethoxazole-trimethoprim.

The C-reactive protein concentration on August 5 was 1.39 mg/dL, and ganciclovir was administered from August 7; however, on August 9, the C-reactive protein concentration had increased to 1.78 mg/dL. Nonetheless, his condition gradually improved to a status of 3/3 CMV antigenemia. Oral findings on August 16, after the start of ganciclovir therapy on August 7, are shown in Figure 4. A persistent ulcer on the left buccal mucosa was noted, and the inflammation was less severe. However, he developed dyspnea on August 18, and blood test results revealed increases in his C-reactive protein concentration and leukocyte count to 24.78 mg/dL and 89.4 × 10^2^/mm^3^, respectively (Figure 5). Computed tomography images of the chest revealed the presence of ground-glass opacities, and noninvasive positive pressure ventilation was initiated under the provisional diagnosis of pneumocystis pneumonia caused by ganciclovir-associated myelosuppression and/or steroid-induced immunocompromised state.

On August 18, his estimated glomerular filtration rate (eGFR) was 25.80 mL/min and blood urea nitrogen (BUN) was 60 mg/dL, while on August 19, eGFR decreased to 19.42 mL/min and BUN increased to 73 mg/dL. A rapid decline in his renal function was evident. Sulfamethoxazole-trimethoprim (10 mL/day) was initiated from August 20. However, the patient died due to respiratory failure on August 25, 2019.

Before his death, the patient provided written informed consent for the publication of his images and case details. The requirement for ethical approval was waived by the appropriate institutional review board.

## 3. Discussion

The potential routes of CMV infection include vertical transmission via the placenta, birth canal, and/or breast milk; horizontal transmission via the saliva, urine, semen, blood, and/or cervical and vaginal discharge; or organ transplantation. In many cases, CMV infection occurs during the perinatal period or infancy, and many infected individuals are asymptomatic [1]. However, the adrenal glands, lungs, gastrointestinal tract, and/or retinas of immunocompromised hosts could be infected. Occasionally, CMV infection may be associated with serious and potentially fatal CMV-related manifestations, such as pneumonia, peptic ulcer, retinitis, and/or encephalitis.

A few reports of intraoral CMV infection [4, 14] in the form of nonspecific ulcers on the lips, palate, tongue, and gingiva [14] have been documented. Moreover, several cases of CMV infection associated with HIV infection, malignant lymphoma, organ transplantation, and autoimmune diseases have been reported.

In contrast, few reports have described oral ulcers caused by CMV in patients with adult nephrotic syndrome, presenting with fatigue, edema, and proteinuria [15], rather than in patients with graft-versus-host disease [16], HIV, or malignant lymphoma. The oral findings in this case included generalized inflamed gingiva and an ulcer on the left buccal mucosa.

A diagnosis of CMV infection can be confirmed by virus isolation; the presence of antigenemia, which detects polymorphonuclear leukocytes that are positive for the viral antigen pp65 [17, 18]; polymerase chain reaction, which detects viral DNA; nucleic acid sequence-based amplification, which detects viral mRNA; and CMV-specific immunoglobulin M antibody analysis. However, in the present case, a histopathological assessment was performed, because infected endothelial cells and fibroblasts could be observed easily in the samples obtained from the ulcer.

In the specimen obtained from our patient, the nuclei and cytoplasm of hematoxylin and eosin-stained cells were positive for the viral antigens, and positive cells were also observed during the immunohistopathological examination. Particularly, vascular endothelial cells show high affinity for CMV and harbor the virus [19]. The infected cells swell and rupture, impeding blood flow and leading to ulceration. Our patient was immunocompromised owing to steroid treatment for membranous nephropathy, and the swelling of the vascular endothelial cells caused by intranuclear inclusions may have led to the circulatory disturbance.

In principle, antiviral medications such as ganciclovir are indicated for CMV infections (e.g., interstitial pneumonia, retinitis, and/or enteritis) [9, 19, 20]. As a result, CMV antigenemia improved from 546/434 to 3/3.

The patient presented with renal dysfunction and developed pneumocystis pneumonia after the onset of the clinical CMV infection. Pneumocystis pneumonia is a serious manifestation of an opportunistic pathogen such as CMV, and previous reports have documented concurrent CMV infection and pneumocystis pneumonia in patients with HIV [21].

The dose and duration of ganciclovir therapy with respect to immune dysfunction were appropriate. The susceptibility of the patient to pneumocystis pneumonia could be attributed to the steroid therapy initiated for the original membranous nephropathy and the myelosuppression as a side effect of ganciclovir therapy.

Our findings also show the relationship between CMV infection and pneumocystis pneumonia. Potentially, the pneumocystis pneumonia in our patient may have been related to the immunocompromised state induced by the steroid use and/or myelosuppression, which is an adverse reaction associated with ganciclovir therapy. Myelosuppression can be prevented by preemptive administration of ganciclovir, which can result in significant dose reduction compared to the normal dose [21].

In conclusion, we report a case of oral ulcer caused by CMV infection in a patient with membranous nephropathy, which was severe and showed atypical manifestations. CMV infections in the oral cavity are not common. However, close collaboration with medical departments is essential when an immunocompromised patient with oral CMV infection is encountered, considering the possibility of a negative outcome.

## Figures and Tables

**Table 1:** The extent of kidney damage in terms of creatinine level of blood, estimated glomerular filtration rate (eGFR), protein-creatinine ratio, uric acid (UA), blood urea nitrogen (BUN), blood sodium, potassium, and chloride levels and serum phosphorus level at the start of treatment, midtreatment (8 days), and posttreatment (18 days).

	8/5/19	8/13/19	8/23/19
	Start of treatment	Midtreatment (8 days)	Posttreatment (18 days)
Creatinine level of blood (mg/dL)	2.0	1.5	3.7
eGFR	25.9	34.6	13.2
Protein-creatinine ratio	3.0	4.4	1.5
UA (mg/dL)	6.3	4.6	13.0
BUN (mg/dL)	54.0	39.0	134.0
Blood sodium level (mEq/L)	125.0	130.0	144.0
Blood potassium level (mEq/L)	4.8	4.6	4.4
Blood chloride level (mEq/L)	96.0	99.0	109.0
Serum phosphorus level (mg/dL)	3.9	2.4	7.9
Urinary creatinine (mg/dL)	105.9	77.6	74.0

## Data Availability

During the course of this research, no data was analysed, reused, or generated.
